# Protein succinylation: regulating metabolism and beyond

**DOI:** 10.3389/fnut.2024.1336057

**Published:** 2024-02-06

**Authors:** Xiaoli Hou, Yiqiu Chen, Xiao Li, Xianliang Gu, Weixia Dong, Jie Shi, Shaoping Ji

**Affiliations:** ^1^Department of Basic Medicine, Zhengzhou Shuqing Medical College, Zhengzhou, China; ^2^Zhoukou Vocational and Technical College, Zhoukou, China; ^3^Department of Biochemistry and Molecular Biology, Medical School, Henan University, Kaifeng, China

**Keywords:** post-translational modification, succinylation, metabolism, regulation, redox

## Abstract

Modifications of protein post-translation are critical modulatory processes, which alters target protein biological activity，function and/or location, even involved in pathogenesis of some diseases. So far, there are at least 16 types of post-translation modifications identified, particularly through recent mass spectrometry analysis. Among them, succinylation (Ksuc) on protein lysine residues causes a variety of biological changes. Succinylation of proteins contributes to many cellular processes such as proliferation, growth, differentiation, metabolism and even tumorigenesis. Mechanically, Succinylation leads to conformation alteration of chromatin or remodeling. As a result, transcription/expression of target genes is changed accordingly. Recent research indicated that succinylation mainly contributes to metabolism modulations, from gene expression of metabolic enzymes to their activity modulation. In this review, we will conclude roles of succinylation in metabolic regulation of glucose, fat, amino acids and related metabolic disease launched by aberrant succinylation. Our goal is to stimulate extra attention to these still not well researched perhaps important succinylation modification on proteins and cell processes.

## Introduction

Protein posttranslational modifications (PTMs) regulate the biological processes of human diseases by cellular pathophysiology regulation and genetic code expansion (1). In recent years, post-translational modifications of protein, such as propionylation, glutarylation, acetylation and succinylation, have become the focus of research (2, 3). Succinylation has emerged as a key regulator of metabolic pathways in various organisms (1, 3–5). This modification involves the transfer of a succinyl group (-CO-CH2-CH2-CO-) from succinyl-CoA to the ε-amino group of lysine residues in target proteins (6–8). The transfer of the succinyl group is catalyzed by succinyltransferases or non-enzyme catalyzed. For the enzymes, they are a family of enzymes conserved across different species (9–11). The non-enzymatic catalysis of succinylation may regulate the succinylation of cytoplasmic protein lysine through the activity of cytoplasmic SIRT5 (7).

Succinyl-CoA is a key intermediate in the tricarboxylic acid (TCA) cycle, a major metabolic pathway that generates ATP and other metabolic intermediates (7). Succinyl-CoA is also involved in other metabolic pathways, including the metabolism of branched-chain amino acids and the biosynthesis of heme (12). Concentration dependence of Succinyl-CoA and resulting proteins modified can be touched on. Both The levels of succinylated proteins and concentration of succinyl-CoA are higher than any other organs of Atrial fibrillation (AF) patients (13).Succinylation has been shown to regulate the activity, stability, and subcellular localization of metabolic enzymes involved in key metabolic pathways (9, 14, 15). For example, succinylation of the TCA cycle enzyme, succinate dehydrogenase, inhibits its activity and impairs mitochondrial respiration, leading to increased production of reactive oxygen species. In contrast, succinylation of the glycolytic enzyme, glyceraldehyde-3-phosphate dehydrogenase, enhances its activity, leading to increased flux through the glycolytic pathway (16–19).

Succinylation has also been implicated in the regulation of mitochondrial function and oxidative stress response (8, 20). Dysregulation of succinylation has been associated with various metabolic disorders (21, 22). For example, decreased succinylation of key mitochondrial enzymes has been observed in the skeletal muscle of individuals with type 2 diabetes (23).

Recent studies have identified a growing number of succinylated proteins in metabolic regulation (3, 24–26). Succinylation is a highly dynamic process responsive to changes in nutrient status and redox state (22, 27). For example, the level of succinylation of key metabolic enzymes has been shown to be regulated by changes in the availability of succinyl-CoA, a key precursor for succinylation (25).

Succinylation also plays a critical role in the regulation of mitochondrial function and oxidative stress response (28). Mitochondrial dysfunction has been implicated in a variety of diseases, including metabolic disorders and neurodegenerative diseases (1, 2, 15, 29). Succinylation has been shown to regulate mitochondrial function by modulating the activity of key enzymes involved in the TCA cycle and oxidative phosphorylation (13). Reactive oxygen species (ROS) are generated during normal cellular metabolism and can cause damage to cells (30). Elevated levels of succinylation in Cancer cell hepatocellular carcinoma are associated with unfavorable survival results (31–33). Peroxisomal fatty acid oxidation，propionate and/or ketone body metabolism that lead to succinyl-CoA production, or some other yet to be discovered pathway encompassing succinyl-CoA, potentially support succinylation of extramitochondrial proteins (6, 7, 34).

### Regulation of succinylation in glucose metabolism

It has been well known that glucose metabolism is a tightly regulated process, and alterations in its regulation can contribute to the development of metabolic disorders such as obesity and diabetes (35). Protein succinylation has emerged as a key player in the regulation of glucose metabolism, by influencing the activity of various enzymes involved in the metabolism (36, 37).

One important enzyme affected by succinylation is pyruvate dehydrogenase complex (PDHC), which is responsible for converting pyruvate to acetyl-CoA. An increase of total protein succinylation promotes degradation of amino acids, which in turn decrease production of acetyl-CoA, leading to decreased glucose utilization and energy production (34, 38). In addition to PDHC, succinylation also affects other enzymes involved in glucose metabolism, including those in the tricarboxylic acid (TCA) cycle and the electron transport chain (39).These modifications can alter the enzymatic activity and the flow of metabolites within these pathways, ultimately impacting glucose utilization and energy production (40).

Recent studies have highlighted specific succinylated proteins involved in glucose metabolism, providing insights into their regulatory roles. For instance, succinylation of fructose-1,6-bisphosphate aldolase has been shown to regulate glycolysis, the initial step in glucose metabolism. Additionally, succinylation of ATP citrate lyase influences the production of acetyl-CoA, a key metabolite in glucose metabolism. It has been discovered that the translocation of Pyruvate kinase M2 (PKM2) to mitochondria under glucose starvation is mediated by succinylation (41).

Furthermore, succinylation has been linked to the regulation of insulin signaling. Succinylation of insulin signaling components can modulate their activity, affecting glucose uptake and utilization (42).

### Succinylation in amino acid metabolism

Protein succinylation plays a crucial role in regulating amino acid metabolism (15). One of the primary ways is by modulating the activity of key enzymes involved in amino acid biosynthesis and catabolism (43, 44). Succinylation has been shown to impact the synthesis of thyroid hormones (45). By succinylating these enzymes, their catalytic activity can be either enhanced or inhibited, thereby tightly and finely controlling the rates of amino acid production and degradation (46).

Succinylation also affects cellular signaling pathways that regulate amino acid metabolism (47). It can affect the activation or inactivation of transcription factors responsible for the expression of genes involved in amino acid synthesis, transport, and utilization. This dynamic regulation ensures that the cell can adapt to changing environmental conditions (48–50).

Furthermore, succinylation is intricately linked to the tricarboxylic acid (TCA) cycle, a central hub in glucose and amino acid metabolism. Succinyl-CoA, an intermediate in the TCA cycle, is a key substrate for succinylation reactions (26, 51). This connection allows succinylation to influence not only energy production but also the availability of carbon skeletons for amino acid biosynthesis (22).

Remarkably, succinylation emerges as a pivotal player in the fine-tuned regulation of amino acid metabolism. It can modify enzymes, modulate gene expression, and interact with central metabolic pathways underscores its importance in maintaining cellular amino acid homeostasis. Understanding succinylation’s role in this context has implications for metabolic research and potential therapeutic significancy in amino acid-related diseases (29).

### Regulation of succinylation in fatty acid metabolism

Succinylation primarily regulates fatty acid metabolism is through modulating the activity of key enzymes involved in lipid synthesis and breakdown (15). The mitochondrial trifunctional protein (TFP) is responsible for the chain-shortening of long-chain fatty acids inside the mitochondria. It is subject to significant succinyl lysine post-translational modifications (PTMs) (52).

Succinylation may enhance or inhibit the catalytic activity of these enzymes depending on different enzymes, leading to finely regulating the process of fatty acid synthesis and utilization within the cell, majorly to provide energy (53, 54). Additionally, succinylation can modify signaling molecules that regulate fatty acid metabolism (55). In response to shifting metabolic demands, it can modulate the activity of transcription factors that govern the expression of a specific set of genes related to lipid metabolism. Lysine acetyltransferase 2A KAT2A, known as GCN5-mediated succinylation of histone H3K79 contributes to the epigenetic regulation of cccDNA minichromosome in HBV, which is important for the regulation of gene expression in tumor cells (11, 48).

Moreover, the link between succinylation and central metabolic pathways, such as the tricarboxylic acid (TCA) cycle, further underscores its role in fatty acid metabolism. Succinyl-CoA, a key intermediate in the TCA cycle, is a substrate for succinylation reactions. This connection not only affects energy production but also influences the availability of carbon precursors for fatty acid synthesis (56).

In conclusion, additional research is essential to fully grasp the significance of succinylation in the realm of fatty acid metabolism. A deeper understanding of how succinylation influences this metabolic process holds the potential to advance our comprehension of metabolic disorders and open doors to potential therapeutic interventions.

### Succinylation in ketone body synthesis

The regulation of succinylation is a crucial factor in controlling ketone body synthesis within the intricate landscape of cellular metabolism. Succinylation emerges as a pivotal player in modulating the production of ketone bodies, essential molecules that serve as an alternative energy source (23, 57), particularly during fasting, low carbohydrate intake, or strenuous exercise (58, 59).

At its core, succinylation regulates ketone body synthesis by influencing the activity of key enzymes involved in this metabolic pathway (60). By adding succinyl groups to specific lysine residues on these enzymes, succinylation can either enhance or inhibit their catalytic function. This precise regulatory mechanism ensures that ketone body production aligns with the body’s energy requirements (61).

Succinylation’s reach extends beyond enzymatic control, encompassing the transcription factors that orchestrate gene expression related to ketone body synthesis. Mutation in the OXCT1 gene caused Succinyl-CoA:3-oxoacid CoA-transferase (SCOT/Oxct1) deficiency in plasma and muscle, which is an inborn error of ketone body utilization characterized by intermittent ketoacidosis crises (23, 60). This modification can modulate the activity of these transcription factors, consequently impacting the expression of genes responsible for ketogenesis, the process of converting fatty acids into ketone bodies. Succinyl-CoA, an intermediate in the TCA cycle, serves as a substrate for succinylation reactions, directly affecting the availability of precursors essential for ketone body synthesis (58).

The regulation of succinylation serves as a vital mechanism in finely tuning ketone body synthesis. Through its influence on enzymes, transcription factors, and integration with central metabolic pathways, succinylation ensures the body’s capacity to produce ketone bodies precisely when needed to meet varying metabolic demands (62, 63). A deeper exploration of this regulatory process holds potential for advancing our understanding of ketone body metabolism and its implications for health and disease (64, 65).

**Figure 1:**
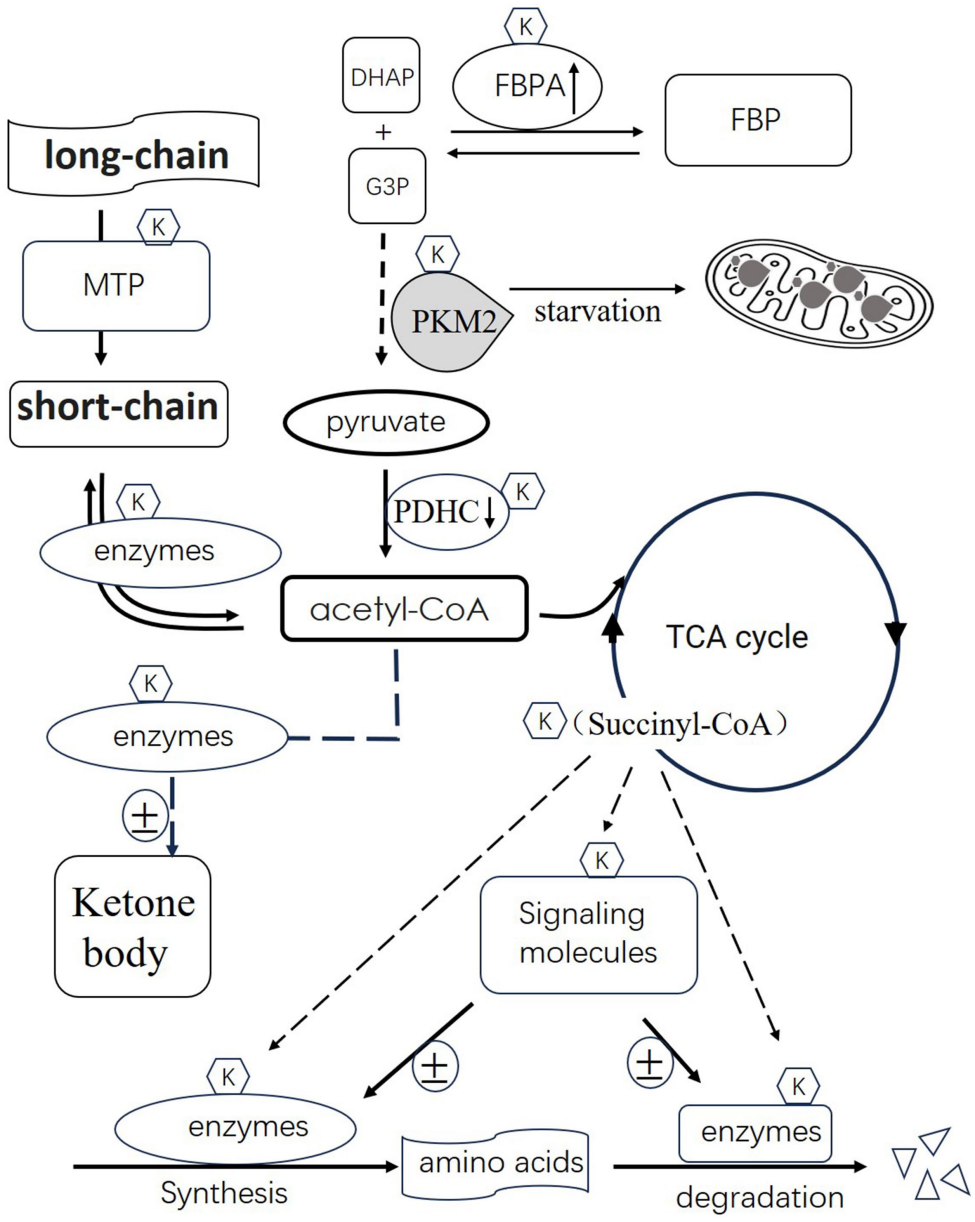
Illustrates the schematic representation of succinylation’s role in regulating metabolism. Succinylation activates 1,6-bisphosphate aldolase (FBPA), enhancing its activity to catalyze the synthesis of dihydroxyacetone phosphate (DHAP) and glyceraldehyde-3-phosphate (G3P) into fructose-1,6-bisphosphate (FBP). Pyruvate kinase M2 (PKM2), crucial in glycolysis, is induced by starvation and undergoes succinylation. This modification facilitates PKM2 translocation into mitochondria. The pyruvate dehydrogenase complex (PDHC), responsible for converting pyruvate to acetyl-CoA, undergoes succinylation, leading to its degradation and consequently reducing acetyl-CoA production. Succinyl-CoA, a product of the tricarboxylic acid (TCA) cycle, serves as a succinyl donor to modify various enzymes involved in amino acid and fatty acid metabolism. Additionally, certain succinylated signaling molecules can either activate or inhibit enzyme activity based on the specific enzymes involved. Furthermore, succinylation regulates the activity of enzymes involved in fatty acid metabolism and ketone body synthesis. In summary, succinylation emerges as a crucial mechanism orchestrating the activity of enzymes across various metabolic pathways, impacting energy production and metabolic homeostasis.

Figure 1 illustrates the schematic representation of succinylation’s role in regulating metabolism. Succinylation, a post-translational modification, plays a pivotal role in modulating key enzymes involved in metabolic processes, thereby influencing energy production. One such enzyme affected by succinylation is fructose-1,6-bisphosphate aldolase (FBPA). Succinylation activates FBPA, enhancing its activity to catalyze the synthesis of dihydroxyacetone phosphate (DHAP) and glyceraldehyde-3-phosphate (G3P) into fructose-1,6-bisphosphate (FBP). Another significant enzyme, pyruvate kinase M2 (PKM2), crucial in glycolysis, is induced by starvation and undergoes succinylation. This modification facilitates PKM2 translocation into mitochondria. The pyruvate dehydrogenase complex (PDHC), responsible for converting pyruvate to acetyl-CoA, undergoes succinylation(one component), leading to its degradation and consequently reducing acetyl-CoA production. Succinyl-CoA, a product of the tricarboxylic acid (TCA) cycle, serves as a succinyl donor to modify various enzymes involved in amino acid and fatty acid metabolism.

The succinylated component of mitochondrial trifunctional protein(MTP) affects processing of long-chain fatty acids to short-chain. The following processing of short-chain to acetyl-CoA is also affected by succinylated enzyme. Additionally, enzymes participating in both amino acid synthesis and degradation can be modified by succinylation. Certain succinylated signaling molecules can either activate or inhibit enzyme activity based on the specific enzymes involved. Furthermore, succinylation regulates the activity of enzymes involved in ketone body synthesis. The outcomes of these modifications on these enzymes are variable and contingent upon their specific metabolic functions. In summary, succinylation emerges as a crucial mechanism orchestrating the activity of enzymes across various metabolic pathways, impacting energy production and metabolic homeostasis.

### Succinylation in redox regulation

Metabolism, particularly glucose metabolism plays a critical role in regulation redox balance. In recent years, research has unveiled how succinylation can intricately modulate redox regulation (27). Firstly, it directly influences the activity of enzymes involved in redox reactions. By succinylating these enzymes, succinylation can either enhance or inhibit their function, thereby affecting the delicate equilibrium between oxidants and antioxidants within the cell. Secondly, succinylation can modulate activity of transcription factors that govern the expression of genes related to redox regulation (66). This modulation can lead to adaptive responses to oxidative stress and alterations in the production of antioxidant molecules (67).

Furthermore, succinylation is intimately connected to central metabolic pathways, particularly the tricarboxylic acid (TCA) cycle, where succinyl-CoA is an essential intermediate. This connection can impact the generation of reducing equivalents like NADH, crucial for maintaining the cellular redox state. In conclusion, succinylation stands as a dynamic and emerging regulator in redox signaling, orchestrating enzyme activity and gene expression to maintain cellular redox balance and adapt to oxidative environments (7) (Table 1).

**Table 1:** Ksucc sites, Location of distribution, Related disease of identified succinylated proteins.

Functional classification	Succinylated protein	Ksucc sites	Location of distribution	Related disease	References
Regulation of glucose metabolism	Histone H4	K5, K12	The germ cells of testes	Male reproductive injury	(16)
	Phosphoglycerate mutase 1(PGAM1)	K99	Cancer cells	Liver cancer	(18)
	lactate dehydrogenase A (LDHA)	k222	Cancer and adjacent tissues	Gastric cancer(GC)	(19)
Regulation of amino acid metabolism	S100A10	K47	Cancer cells	GC	(46)
	fibrillin 1(FBN1)	k672	Cancer cells	GC	(47)
	kidney-type glutaminase (GLS)	K311	blood serum and Cancer cells	metabolic diseases and cancer	(22, 44)
Regulation of fatty acid metabolism	cardiac proteins:ECHA		heart	hypertrophic cardiomyopathy,	(53)
	enoyl-CoA hydratase (EchA19)	K132, K139	*Mycobacterium tuberculosis* (Mtb)	infection	(54)
Regulation of ketone body synthesis	3-ketoacid-CoA transferase(SCOT)	/	Skeletal muscle	hyperglycemia	(23)
	cardiac protein O-GlcNAcase	/	diabetic myocardium	type 2 diabetes mellitus and heart failure	(61)
	The ketoglutarate dehydrogenase complex (KGDHC)	/	Brain(all cortical cells)	neurodegenerative diseases	(6)
Redox regulation	Pyruvate kinase M2 (PKM2)		Cancer cells	colon cancer	(41)
	LDHA and SDHA		vascular smooth muscle cells	aortic aneurysm and dissection (AAD)	(20)
	GTPase Cdc42		serum and brain tissue	occlusion of the middle cerebral artery	(30)
Others	Nucleosomal histone H4	K77	tumor cells	DNA repair defect	(50)
	Nucleosomal histone H3	K79, K122	Liver cells	HBV-infected	(11, 68)
	CTBP1	K46, K280	Cancer cells	prostate cancer	(63)
	Influenza virusviral nucleoprotein	K87	lung	Influenza virus infection	(49)
	HCMEC/D3 protein (TAGLN2)	K40	Tumor cells	gliomas	(33)

## Perspective

The regulation of succinylation in metabolism represents a captivating frontier in our understanding of cellular homeostasis. This post-translational modification has emerged as a key player in fine-tuning metabolic processes.

Succinylation exerts its influence across diverse metabolic pathways, impacting enzymatic activities, gene expression, and central metabolic hubs like the tricarboxylic acid (TCA) cycle. This interconnectedness underscores its significance in modulating energy production, amino acid metabolism, fatty acid synthesis, and redox regulation.

Moreover, succinylation’s responsiveness to environmental cues, such as changes in nutrient availability or oxidative stress, highlights its role as a metabolic sensor. It enables cells to adapt swiftly to varying conditions, optimizing energy utilization and metabolic flux.

Understanding the intricate network of succinylation in metabolism holds immense promise. It may unveil novel therapeutic targets for metabolic disorders like diabetes, obesity, and cancer. Moreover, it offers insights into broader aspects of cellular health, aging, and disease. As researchers delve deeper into this fascinating field, more attention should be given to the reversible succinylation of lysines within mitochondria, as well as its potential application in anticancer drugs will be provided. we anticipate remarkable revelations that will reshape our comprehension of metabolism and its impact on human health.

## Author contributions

XH: Data curation, Formal analysis, Resources, Writing – original draft. YC: Conceptualization, Data curation, Writing – original draft. XL: Resources, Writing – original draft, Methodology. XG: Writing – original draft, Methodology, Resources. WD: Writing – original draft, Formal analysis. JS: Formal analysis, Validation, Writing – original draft. SJ: Conceptualization, Funding acquisition, Supervision, Writing – review & editing.
